# Online Contingent Attention Training (OCAT): transfer effects to cognitive biases, rumination, and anxiety symptoms from two proof-of-principle studies

**DOI:** 10.1186/s41235-023-00480-3

**Published:** 2023-05-08

**Authors:** Ivan Blanco, Teresa Boemo, Oscar Martin-Garcia, Ernst H. W. Koster, Rudi De Raedt, Alvaro Sanchez-Lopez

**Affiliations:** 1 Department of Clinical Psychology, Complutense University of Madrid, 28223 Madrid, Spain; 2 Department of Experimental Clinical and Health Psychology, Ghent University, 9000 Ghent, Belgium

**Keywords:** Attention bias modification, Interpretation bias modification, Smartphone app, Emotion regulation, Psychological symptoms

## Abstract

The aim of the present research was to develop and test the efficacy of a novel online contingent attention training (i.e., OCAT) to modify attention and interpretation biases, improve emotion regulation, and reduce emotional symptom levels in the face of major stressors. Two proof-of-principle studies were carried out. In study 1, 64 undergraduates who were about to start a major stressful period (i.e., final exams) were randomized to undergo 10 days of active OCAT or a sham-control training. Emotion regulation (habitual use of rumination and reappraisal) and symptom levels (depression and anxiety) were assessed before and after the intervention. In study 2*,* the same 2 × 2 mixed design was used with 58 individuals from the general population undergoing a major stressful situation (the lockdown period at the beginning of the COVID-19 pandemic in 2020). In both studies, the OCAT group showed significant improvements on attention towards negative information and interpretation biases in comparison to the sham-control group. Additionally, changes in cognitive biases transferred to reductions of participants’ use of rumination and anxiety symptom levels. These results show preliminary evidence regarding the efficacy of the OCAT to target attention and interpretation biases as well as to improve emotion regulation processes and to buffer against the effects of major stressors.

**Supplementary Information:**

The online version contains supplementary material available at 10.1186/s41235-023-00480-3.

## Significance statement

Cognitive biases (e.g., attention and interpretation biases) are conceptualized as central mechanisms for the onset and maintenance of emotional disorders such as depression and anxiety. Cognitive models have posited that these biases emerge under stressful situations, impairing peoples’ ability to regulate negative emotions, ultimately leading to the appearance of emotional symptoms. Given the current worldwide prevalence of emotional disorders and emotion dysregulation problems, there is a clear need for new approaches targeting these cognitive biases at a broad and accessible scale.

In the present two proof-of-principle studies, we adapted a well-established cognitive biases modification paradigm (CBM) into a novel smartphone app (OCAT) for training participants’ attention and interpretation biases during two different naturalistic stressful situations. In Study 1, undergraduate students completed the OCAT training (10 session per day) when they were facing their final examination period. In Study 2, people from the community completed the same training during the occurrence of a very restrictive COVID-19 lockdown. Overall, these studies showed the efficacy of the OCAT app to modify both attention and interpretation biases, as well as transfer effects to improve emotion regulation and reduce emotional symptoms.

These findings shed light regarding the causal role of cognitive biases on emotion regulation and emotional symptoms. Moreover, provide further encouragement for the further development and dissemination of CBM paradigms that can be applied in online formats.

## General introduction

Depression and anxiety disorders are highly prevalent worldwide (Steel et al., 2014) and their impact and burden, both in terms of individual suffering and societal and economic costs, are increasing during the last decades (Jorm et al., 2017). Notwithstanding the wide range of evidence-based treatments that are available for these common mental disorders, their accessibility and efficacy have often been questioned. First, epidemiological studies show that access to adequate psychological treatment is still too limited (Barbato et al., 2016). Second, existing interventions have shown limited efficacy in both recovery and relapse prevention (Cuijpers et al., 2010, 2020). These data clearly show the need for new approaches to reduce the incidence and prevalence of common mental disorders. In this sense, the NIMH pointed out that one major strategy to achieve this goal is translating the emerging findings of experimental research and neuroscience into novel fine-grained psychological treatments that target specific psychological mechanisms underlying mental disorders (NIMH, 2011).

Importantly, novel approaches conveying the use of precise knowledge on neurocognitive mechanisms of stress and emotional dysfunctions have been developed in recent years (see, for instance, Goodwin et al., 2019). Among those mechanisms, experimental research indicates that emotional disorders are characterized by biased information processing (i.e., preferential attention to, interpretation of and memory for negative over positive information; see Mathews & MacLeod, 2005). Accumulating empirical evidence shows that depressed and dysphoric individuals preferentially attend to negative emotional information, and significantly less to positive information, in comparison to healthy or non-dysphoric individuals (e.g., Duque & Vázquez, 2015; Blanco et al., 2019). Furthermore, depressed and dysphoric people show more negative than positive interpretations when they are processing ambiguous information (Everaert et al., 2017). Similar results have been found in individuals with anxiety. In terms of attention biases, previous empirical research supports that participants with both clinical and sub-clinical anxious symptoms show preferential attention towards negative information in comparison to healthy control participants (Armstrong & Olatunji, 2012; Pergamin-Hight et al., 2015;). Additionally, research has also shown that anxiety is characterized by problems to disengage attention from negative information (Fox et al., 2002; Wang et al., 2019). Such attentional disengagement difficulties have also been found in depressed participants (see Sanchez et al., 2013). Regarding interpretation biases, cognitive models posit that negatively biased misinterpretation of information (i.e., interpretation biases) might also be a relevant factor implicated in the onset and maintenance of anxiety disorders (Mathews & MacLeod, 1994). This negative interpretation bias is not only restricted to anxiety disorders such as social anxiety or generalized anxiety disorder (see Hirsch et al., 2016), but it is also present in participants with a heightened vulnerability to develop anxiety disorders (Hirsch & Mathews, 1997). Taken together, processing information in a negatively biased manner seems to be a transdiagnostic factor for both forms of psychopathology (Garland & Howard, 2014).

These attentional and interpretation biases are interrelated (Everaert et al., 2014), and are thought to be causally involved in the emergence of emotional symptomatology through their role in the generation of emotion regulation impairments such as a high use of rumination and/or a less efficient use of reappraisal strategies (Joormann & Vanderlind, 2014). Specifically, such emotion regulation impairments resulting from negative biases would act as a core risk factor for the development of emotional problems such as depression and anxiety in the face of major stressors (Liu & Alloy, 2010).

Relatedly, recent research has shown that during the COVID-19 lockdown of 2020, a clearly major stressful situation, interpretation and attention biases had a large impact on psychological adjustment to the situation (Blanco et al., 2021). This suggests that attention and interpretation biases play a crucial role in the onset and maintenance of emotional disorders (Mathews & MacLeod, 2005). Hence, the development and empirical validation of new procedures capable of modifying biases in attention and interpretation processes is needed.

During the last two decades, a growing body of research under the umbrella term of attention bias modification (ABM) has emerged, developing procedures to train and modify attention biases related to emotional dysfunctions (Koster et al., 2009). The first generation of ABM procedures used simple behavioral tasks to retrain attention in anxious and depressed individuals. Although initial results were promising, most recent work indicates that these initial ABM procedures might only have limited effectiveness (Cristea et al., 2015; Fodor et al., 2020). Fortunately, recent conceptual and technological advances in the development of eye-tracking technology have opened venues for a new generation of ABM research, where attention is trained through eye-tracking based paradigms and techniques (Lazarov et al., 2017; Price et al., 2016; Shamai-Leshem et al., 2021). These procedures allow researchers to overcome many of the limitations of the previous paradigms. For instance, monitoring attentional patterns through eye-tracking devices allows not only assessing but also training specific dysfunctional attentional components involved in emotional dysfunctions (e.g., maintained attention towards negative information and/or reduced attention to positive information), by introducing online feedback based on participants’ actual attentional performance (Vazquez et al., 2016).

A particularly promising approach is the (eye)gaze-contingent feedback training (ECAT; Sanchez et al., 2016). In the ECAT, participants’ gaze patterns are monitored using eye-tracking technologies while performing a scrambled sentence task (i.e., SST; Wenzlaff & Bates, 1998). The standard SST consists of mentally unscrambling a series of 6-scrambled-words that are displayed on the screen (e.g., “born loser am I winner a”). Participants are asked to unscramble the words to form a grammatically correct sentence using only 5-words. The only two possible solutions to resolve the sentence are either a positive or a negative sentence (e.g., “I am a born winner” or “I am a born loser”, respectively). This task was modified to allow to measure interpretation biases while gaze patterns are being monitored to index attention biases (i.e., time attending to negative vs. positive words) subserving interpretations’ generation (Everaert et al., 2014; Sanchez et al., 2015). In ECAT, participants in the control group are asked to simply unscramble words into the sentence that first come to their mind, whereas participants in the active attention training group are explicitly instructed to shift and maintain their attention towards the positive word, to unscramble the sentences in a more positive manner, thus training both attention and interpretation biases. Further, when participants fixate their gaze on the positive or negative words, a green or red frame appears surrounding the corresponding word, respectively, providing immediately online feedback on their attentional patterns. Therefore, gaze-contingent feedback and explicit instructions allows participants to increase awareness of attentional biases and to regulate their attention (i.e., top-down regulation) to modify specific biases associated to emotional dysfunctions (i.e., reducing the processing of negative stimuli and shifting and maintaining attention toward positive information) while positive interpretations are being generated.

Using this ECAT, it has been found that, in comparison with the control group, the training group showed less attention bias (AB) to negative information (measured by independent dot-probe and eye-tracking tasks) and that AB changes led to improvements in participants’ reappraisal abilities, to reductions in state rumination, and ultimately to fewer negative emotions during an emotion regulation task (Sanchez et al., 2016; Sanchez-Lopez et al., 2019b). This approach is a promising intervention tool for targeting attention and interpretation biases for depressed and anxious individuals for several reasons. First, this paradigm directly targets emotional biases that are relevant in both types of emotional disorders, included attentional maintenance towards negative information and avoidance of positive information (Armstrong & Olatunji, 2012) and tendencies to interpret ambiguous information on a negative manner (Hirsch et al., 2016). Second, the explicit instructions and gaze-contingent feedback procedures used in these trainings might also target other crucial top-down attentional control strategies (i.e., attentional inhibition and shifting) that are known to be impaired in emotional disorders (Miyake & Friedman, 2012). Third, transfer effects to reappraisal and rumination are crucial for emotional disorders, given that maladaptive use of these emotion regulation strategies are implicated in these disorders (Aldao et al., 2010).

Despite the potential of this training approach, the necessity of advanced eye-tracking techniques for delivery of the ECAT may limit its use as a clinical intervention. Therefore, a new variant of this training, the mouse-based contingent attentional training (MCAT) was developed (Sanchez-Lopez et al., 2019a). This variant has revealed the possibility of training interpretation and attention without the direct use of eye-tracking devices but maintaining the same training principles of the original ECAT approach. As in the original ECAT, participants in the training group are asked to unscramble the emotional sentences in a positive manner. However, in MCAT, words within the scrambled sentence appear hidden on the screen, and participants must move the mouse-pointer over the screen to uncover the words, receiving mouse-contingent feedback similar to the ECAT feedback when positive and negative words are unhidden/attended. Interestingly, Sanchez-Lopez et al (2019a) conducted eye-tracking in parallel to the performance of this task variant, with results showing a high correlation between the real gaze patterns and the attention patterns indexed though the movement of the mouse-pointer over the screen. This supports the validity of the mouse-based attention estimations. Furthermore, the training results using the MCAT variant were similar to those using the original ECAT, with trained participants showing less AB towards negative information, more use of reappraisal and less use of rumination in response to negative events than those ones in the control group; Sanchez-Lopez et al., 2019a). Yet, this new procedure was developed with dedicated experimental software (such as E-Prime) and requires using computers to deliver the training. This can still limit the feasibility of the training to be fully implemented online and its large-scale dissemination to individuals who are suffering (or in risk of having) emotional disorders in conditions of major stress.

Therefore, the main aim of the present research was to examine the efficacy of a new variant, the Online Contingent Attentional Training (OCAT), developed as a fully accessible online app that can be executed either on computers or on mobile phones. The novel OCAT variant follows the exact same principles used in MCAT and is designed to modify attention and interpretation biases and transfer to improve emotion regulation and emotional symptomatology. Across a series of two proof-of-principle studies, a 10-session version of OCAT was validated, analyzing its transfer effects to the use of emotion regulation strategies (i.e., rumination and reappraisal) and to emotional symptomatology levels (i.e., depression and anxiety). Accumulating research has shown that mechanisms of vulnerability to emotional dysfunctions such as attention and interpretation biases can emerge and contribute to dysfunctions particularly at conditions of major stress (see, for instance, Everaert et al., 2012). In this respect, it is important to be able to modify these biases as they manifest during naturalistic stress conditions. The new OCAT was designed with this aim in mind. Thus, for OCAT validation purposes, the present two studies were conducted using different populations under different conditions of ecological stress. In study 1, conducted in 2019, we analyzed the efficacy of the OCAT training in an unselected sample of undergraduate students facing the beginning of final exams (e.g., Robotham & Julian, 2006). In study 2, we studied the efficacy of OCAT in the general population when facing a major stressful situation (i.e., the COVID-19 lockdown occurring during beginning of 2020).

## Study 1. Effectiveness of OCAT to target cognitive biases, emotion regulation and psychological symptoms during a stressful exam period in undergraduate students

The main aim of this first study was to validate the new OCAT variant and analyze its efficacy as a 10-session online protocol to modify both attention and interpretation biases and transfer to improvements in the use of emotion regulation strategies and/or emotional symptomatology. Following previous research (Sanchez-Lopez et al., 2019a), we hypothesized that trainees would show more attention to positive information, more positive interpretations, as well as increased use of reappraisal and lower use of rumination in comparison to the control group at post-training. Additionally, it was hypothesized that further transfer effects of active but not control OCAT would be observed at the level of symptom improvement (i.e., lower levels of depression and anxiety symptoms in the face of the stressor at post-training.

## Method

### Participants

**Fig. 1:**
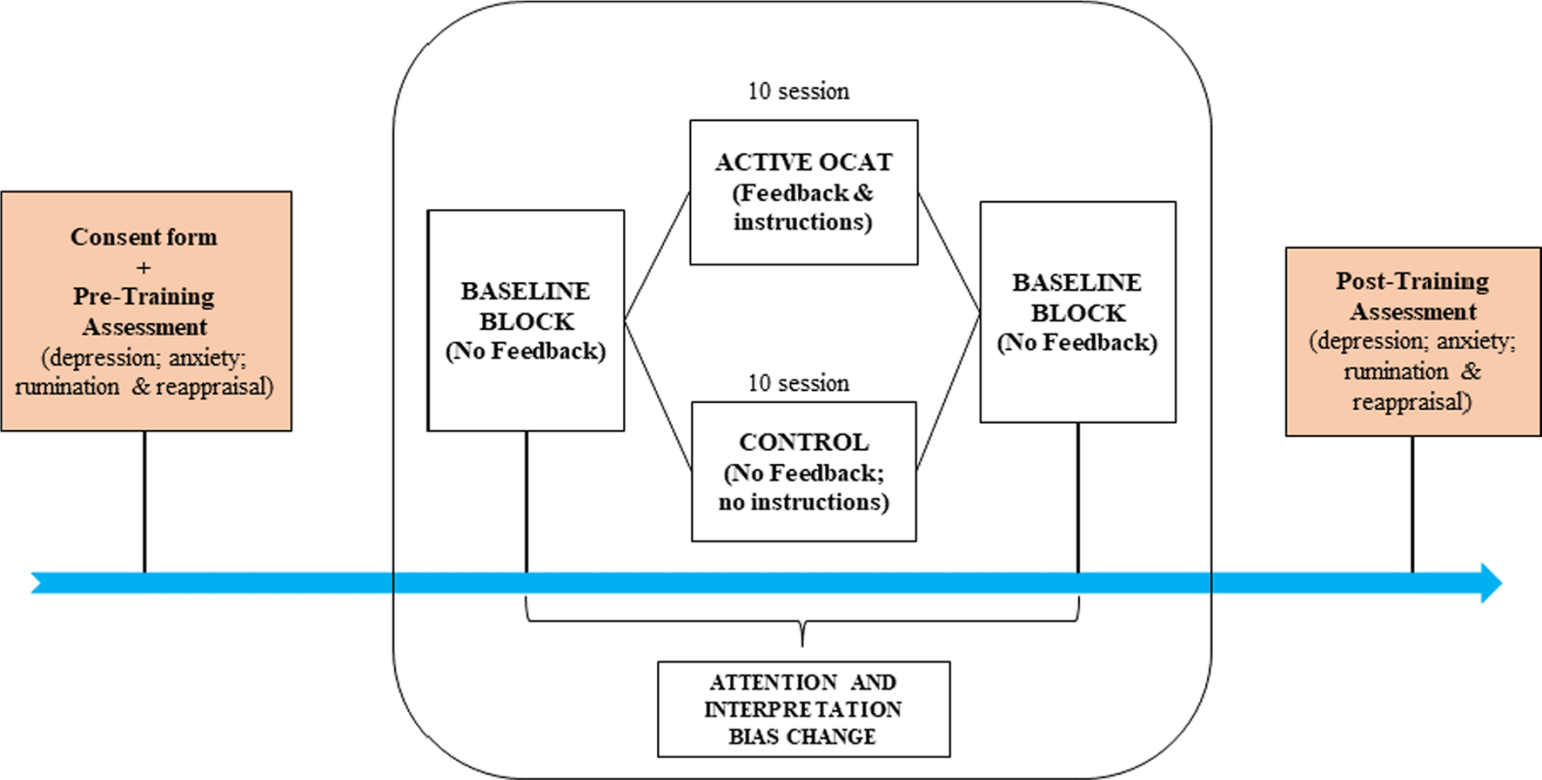
General procedure

Sixty-four undergraduate students took part in the study in exchange for course credits. All participants were recruited three-weeks before they began their final examination period and they were then randomly allocated to start the active OCAT or the control sham-training group in the same time period (i.e., within the same week). A simple randomization procedure, where all participants had the same probability of being allocated to the active OCAT or the control sham-training group, was carried out. This procedure was performed through a macro excel file which automatically assign the participants to one of the two experimental conditions. Nine participants of the active OCAT group and 5 of the control sham-training group were excluded from the analysis due to dropout and/or technical issues. Additionally, 2 participants were identified as outliers, defined as having scores ± 2.5 SD on the baseline assessment of the main outcome (i.e., attentional bias index), and were excluded (see Additional file 1: Appendix A for the full flow diagram of participant’s recruitment and allocation). The final active OCAT group was composed of 22 participants (68% female; mean age = 21.5 years—*SD* = 1.94) whereas 24 participants were in the control sham-training group (67% female; mean age = 21.65 years—*SD* = 2.4) (see Fig. 1 for full details). All participants signed an informed consent form before starting their participation in the study.

### General procedure

As depicted in Fig. 1, after signing the informed consent form and completing the pre-training psychological measures, baseline attention and interpretation biases were assessed on the smartphone app through an SST variant similar to the one used in the MCAT procedure (see Sanchez-Lopez et al., 2019a). Immediately afterwards, participants completed the first session of the active or control sham-training in the laboratory. The remaining nine training sessions were completed daily, online (1 session per day). Participants in both conditions were recommended to complete daily training sessions in a quiet environment without distractions and, when possible, at moments that they felt particularly stressed during the day. At the end of the last tenth session, attention and interpretation biases were assessed again with the SST and, the day after, participants completed the post-training psychological assessments (see Fig. 1). The study was conducted in compliance with the Declaration of Helsinki (World Medical Association, 2013) and was approved by the University Ethical Committee.

### Experimental task and materials

*SST Stimuli* A total of 300 six-word scrambled sentences covering 7 different topics typically involved in mood and anxiety disorders (i.e., experience of positive and negative affect, cognitive and emotion regulation deficits, neuroticism, health conditions/concerns, self-perception, others/world appraisals, and beliefs/concerns about future) were used in the SST. These unscrambled sentences were divided into 5 different blocks (60 unscrambled sentences per block) controlling for the presence of the same number of sentences of each topic on each block. The emotional words (i.e., positive and negative word) of each sentence were equated, within each topic and block, on length [*F* (24, 265) = 1.5; *p* = 0.070], arousal [*F* (24, 245) = 0.863; *p* = 0.652] and valence magnitude [*F* (24, 245) = 1.04; *p* = 0.414] following normative data (Fraga et al., 2018).

*OCAT procedure* The 10 session OCAT procedure was an adaptation of the previously validated single session MCAT procedure (Sanchez-Lopez et al., 2019a). In that procedure, a variant of the SST (Wenzlaff & Bates, 1998) was used to measure, and train, interpretation and attention biases conjointly through the position and coordinates of the mouse cursor in the computer screen during performance of the task. In OCAT, using the same SST format and following the same principles of action of the MCAT, we used the position and coordinates of participants’ finger on the screen of the mobile phone to both measure and train attention and interpretation biases.

**Fig. 2:**
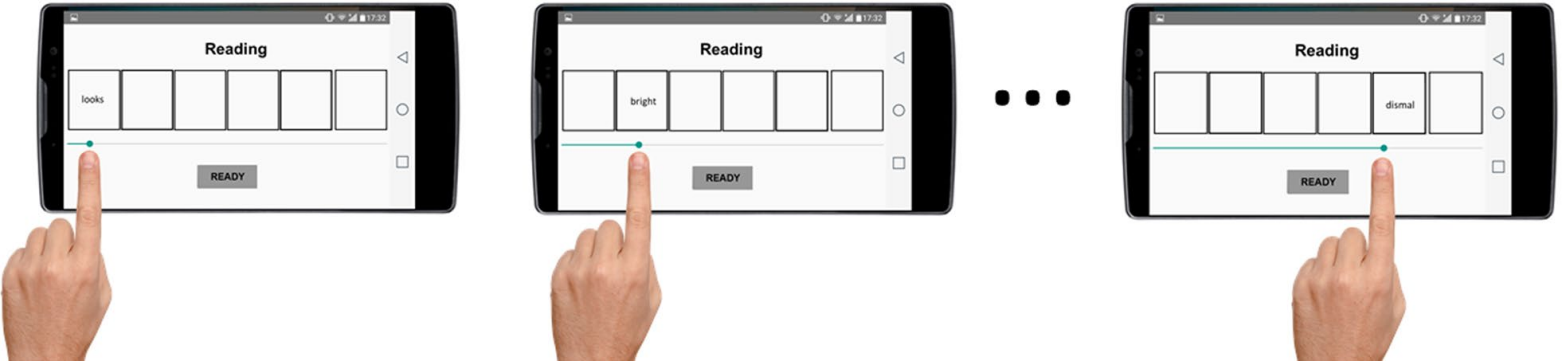
Example of the reading phase during the evaluation phase

*Evaluation phase* Immediately before and after the training phase, all participants performed an evaluation phase of the SST to assess pre-post changes in attention and interpretation biases. These blocks comprised a series of 12 emotional scrambled sentences. The number of trials was established based on previous extensive piloting of sufficient required SST trials to obtain reliable cognitive bias indices related to stress vulnerability and emotional status (Martin-Romero et al., 2023). Noteworthy, this design has proven to derive reliable indices of attention and interpretation biases with predictive power to account for individual differences in emotion regulation use and psychological adjustment to major stress (Blanco et al., 2021). Each scrambled sentence was composed of 6 words (e.g., “looks bright very the dismal future”) that could be unscrambled with a positive (e.g., “the future looks very bright”) or a negative emotional meaning (e.g., “the future looks very dismal”). In the first baseline session, all participants were instructed to unscramble them, as fast as possible, to form the grammatically correct and meaningful sentence that first came to their mind, using only five out of the six words. Each trial started with a fixation cross at the left size of the phone screen. Participants were asked to press the fixation cross with their finger to start the reading section of the SST. In the *reading section*, and following a moving window technique (Fang et al., 2017), the 6 words were hidden into individuals squares and participants had to move their fingers (using a scroll bar positioned just below the squares) to uncover and read the corresponding word. Once participants moved their fingers away from one word to the position of another word, the previously selected word was hidden again, and the new word was shown (see Fig. 2). This task allows to monitor the exact time that participants were attending and reading each word. Additionally, the emotional words (i.e., positive or negative words) were always positioned in the second and fifth place in order to avoid the influence of word positioning on attentional patterns and subsequent interpretations. The position of positive and negative words was pseudo-randomly assigned, controlling that they were positioned half of the trials in the second position and the other half in the fifth place in each training block.

**Fig. 3:**
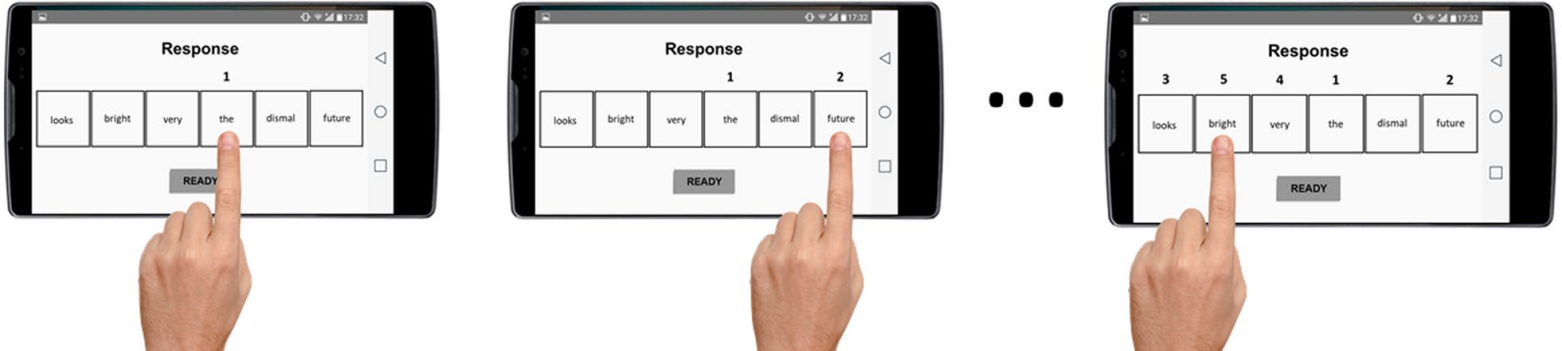
Example of the response phase

After a time-limit of 14 s, or when participants pressed the button “ready” at the bottom of the screen, the *response phase* started. In this phase, all the words were uncovered, and participants had 7 s to compose the mentally unscrambled sentence by pressing in the appropriate order the corresponding 5 words to create the grammatically correct sentence. A number (from 1 to 5) was displayed above each word, pointing to the order in which participants had selected each word. If participants made any mistake during the construction of the sentence, they could modify it by unselecting the wrong word (i.e., removing the number above that word) and selecting the chosen one. The procedure allows to categorize each unscrambled sentence as a positive or negative interpretation (see Fig. 3).

**Fig. 4:**
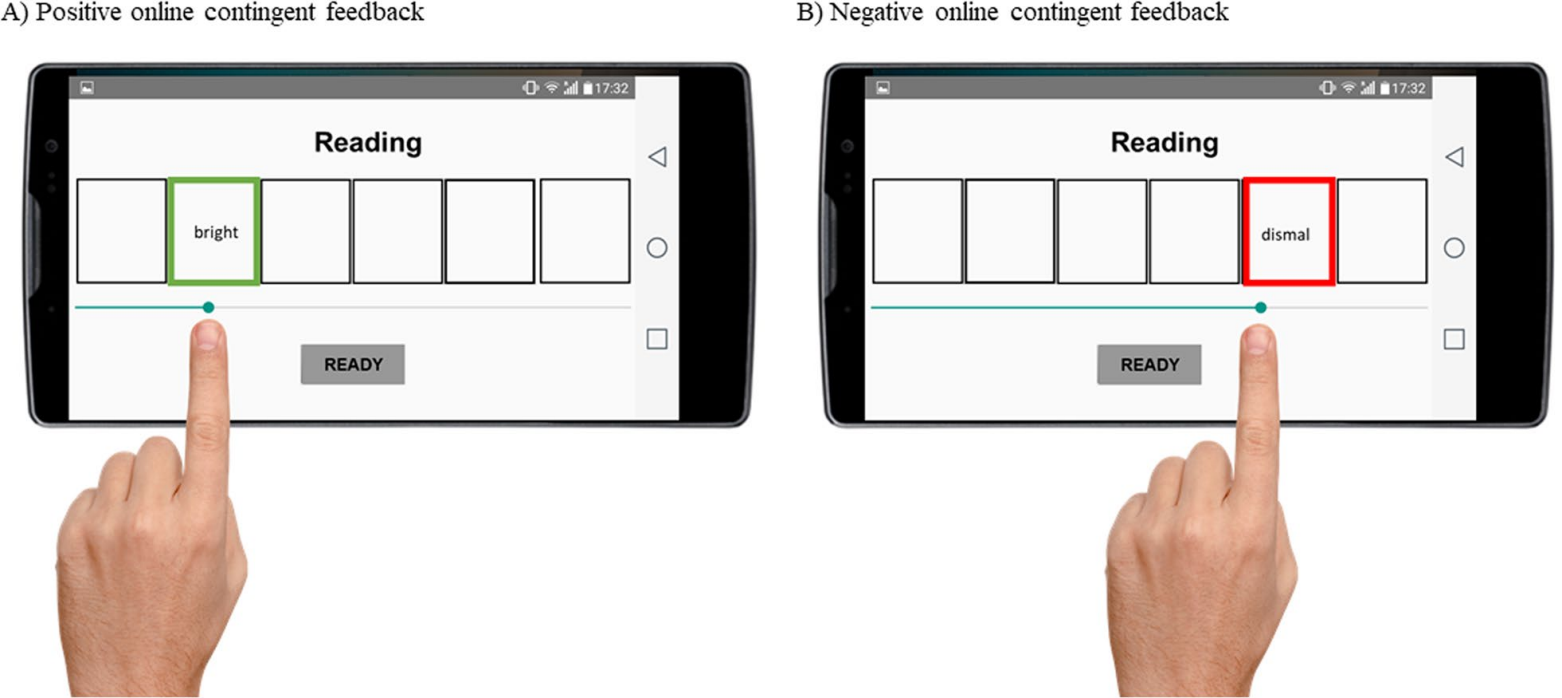
Example of the online contingent feedback during the reading phase of the OCAT active training

*Training phase* Each of the 10 training sessions comprised 8 training blocks of 6 trials each. Participants in the active OCAT group were first instructed to unscramble the sentences, as fast as possible, in a grammatically correct way with a *positive emotional meaning*. As for participants in the control sham-training, they were asked to unscramble the sentences as in the baseline assessment: as fast as possible, reporting the grammatically correct sentence that first comes to mind. Second, participants in the active OCAT condition received online contingent attention feedback during the reading phase. That is, when participants were attending and reading each word, a green or a red square appeared framing the positive or the negative word respectively, each time that any of those words were uncovered by the participants. Trainees were instructed to use this online contingent feedback to disengage attention from the negative words and to focus on processing the positive words (see Fig. 4). Participants in the control sham-training group did not receive any online contingent feedback while performing the task.

**Fig. 5:**
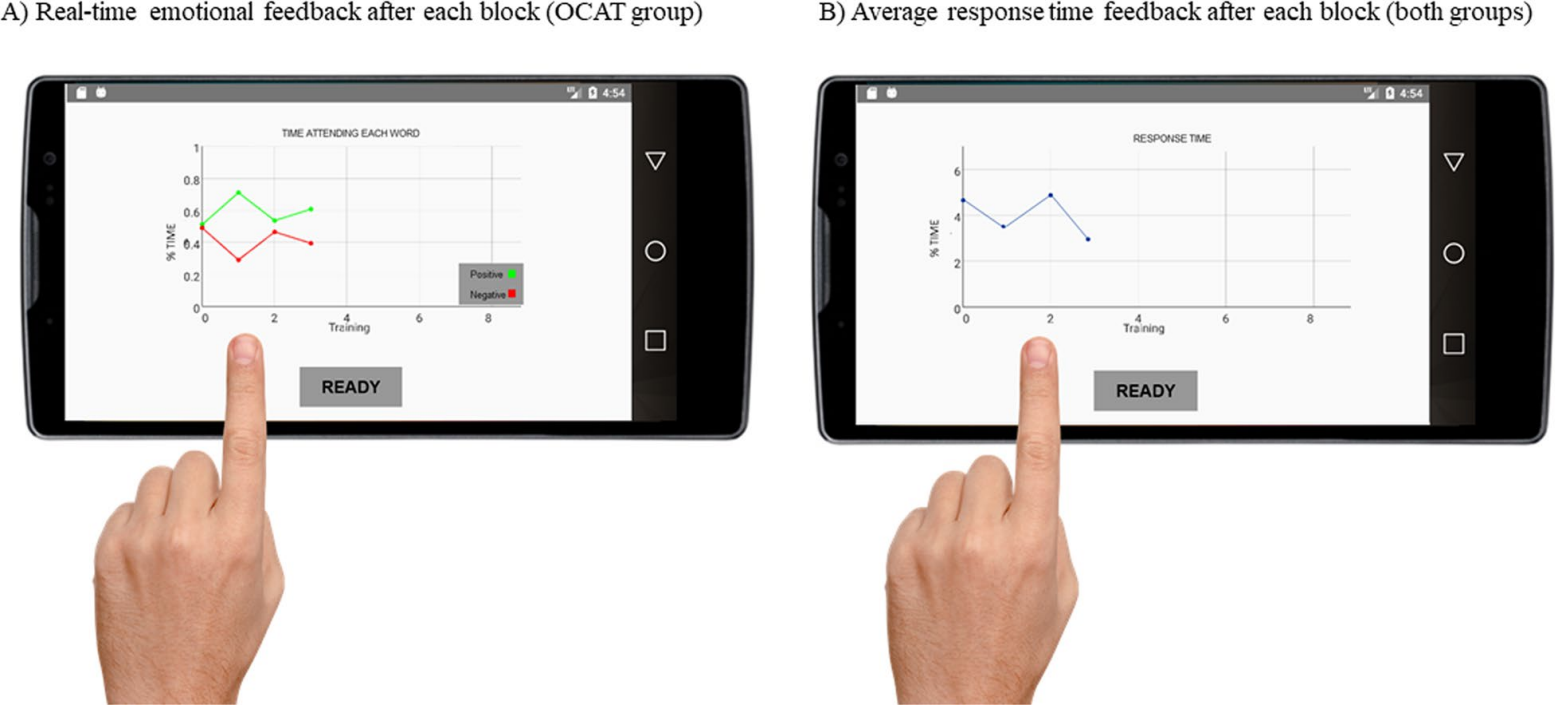
Example of real-time feedback after each block

After each training block, participants in the active OCAT condition received feedback regarding the average time they had been attending to the positive and the negative words in the reading phase of the preceding blocks and, also, regarding their average response time to report the unscrambled sentences in the response phase. They were instructed to use this real-time feedback to increase, from block to block, the time that they had spent attending positive words, and to decrease their response times as well as the time they had spent attending the negative words (see Fig. 5). Participants in the control sham-training group only received feedback regarding their average response time to report unscrambled sentences.

These manipulations (i.e., explicit instructions, online and blockwise feedback about emotional information processing) allow trained participants to increase awareness of their attentional deployment and to use top-down attention regulation strategies (see Vazquez et al., 2016). Furthermore, this design also ensures that the training phase of both groups, active OCAT and sham-control group, only differed in the emphasis on emotional aspects of the training (Blackwell et al., 2017). (A further full visual depiction of both the assessment and modification sections of the active OCAT can be found in Additional file 1: Appendix B).

### Attention and interpretation bias dependent variables

Both mobile apps (i.e., active OCAT and sham-control app) allowed to compute several dependent variables regarding participants’ attention and interpretation biases.

*Attentional processing* To analyze attentional processing, the app registered the total time (in milliseconds) that participants spent uncovering positive and negative words during the *reading phase*. This allowed to analyze specific theory-driven aspects of emotional attention: a) *total time attending positive words*; b) *total time attending negative words*. Cronbach’s alpha was calculated for each measure of attentional processing at both times (before and after the training). Results showed very good to excellent reliability for all these measures (see Table 1).

**Table 1:** Reliability analysis for attention and interpretation biases measures at Time 1 and Time 2

	Time 1	Time 2
Attention measures (α)		
Total time attending positive words	.87	.95
Total time attending negative words	.86	.94
Interpretation biases (λ)	.93	.92

α = Cronbach’s alpha; λ = Guttman split-half coefficient

*Interpretation biases* To analyze interpretation biases, the apps registered the number of positive and negative grammatically correct sentences created by the participants during the *response phase.* This allowed to compute a *positive interpretation index* by dividing the total number of positive grammatically correct sentences by the total number of grammatically correct sentences at pre- and post-training. Split-half reliability analysis were carried out to assess interpretation bias measures’ consistency at each time (before and after the training). Data showed excellent reliability (see Table 1).

### Psychological transfer measures

A series of psychological measures were used to analyze the transfer effects of the active OCAT compared to sham-training from Time 1 to Time 2.

*Depression levels.* The Center for Epidemiological Studies on Depression-8 (CESD-8, Turvey et al., 1999) was used to assess participants’ depressive symptomatology. It is composed of 8 Likert-scale items (ranging from 0- none or almost none of the time to 3- all or almost all the time). Participants select how much of the time (during the past week) they have experienced depressive-related symptomatology (i.e., *feeling depressed; lonely; *etc*.*). Validation studies have shown a good internal reliability of the scale (α = 0.79; Turvey et al., 1999). In our study, the scale showed also a good internal consistency at both Time 1 (α = 0.80) and Time 2 (α = 0.85).

*Anxiety levels* The Generalized Anxiety Disorder Scale (GAD-7; Spitzer et al., 2006) was used to evaluate participants’ general anxiety levels. It comprises 7 Likert-scale items ranging from 0 (not at all sure) to 3 (Nearly every day). Participants select how often they have been bothered by general anxiety-related symptoms such as *becoming easily annoyed or irritable, not being able to stop or control worrying* or *worrying too much about different things*. In the validation study, the scale showed excellent reliability (α = 0.92; Spitzer et al., 2006). In our study, internal consistency was good at both Time 1 (α = 0.82) and Time 2 (α = 0.87).

*Brooding rumination* The subscale of brooding rumination from the Ruminative Response Scale (Nolen-Hoeksema & Morrow, 1991) was used to assess participants’ brooding rumination by 5 Likert-scale items (ranging from 1-almost never to 4- almost always). In the original study, this scale showed good reliability (α = 0.89; Nolen-Hoeksema & Morrow, 1991). In our study, the scale showed a relatively low internal consistency at Time 1 (α = 0.65), although it had adequate internal consistency levels at Time 2 (α = 0.76).

*Positive reappraisal* The reappraisal subscale from the Cognitive Emotion Regulation Questionnaire (CERQ—Garnefski et al., 2002) was used to assess participants’ tendency to use reappraisal as an emotion regulation strategy. This subscale is composed by 4 Likert-scale items ranging from 1 (almost never) to 5 (almost always). The validation of this scale showed that the positive reappraisal scale had good reliability (α = 0.85; Garnefski et al., 2002). Its internal consistency in our study was good at both Time 1 (α = 0.84) and Time 2 (α = 0.87).

Of note, all psychological variables were framed into the previous week (i.e., *during the last week, including today…”*) to maximize their sensitivity to capture changes on psychological and emotion regulation variables as a result of training.

### Data analysis plan

Prior to the analysis, normality and homoscedasticity^1^ were checked through Shapiro–Wilk and Levene tests, respectively. First, to analyze the effects of active OCAT vs. control sham-training on pre-post changes in attentional processing, a 2 Group (OCAT; Control) × 2 Time (Time 1: pre-training; Time 2: post-training) × 2 Type of Stimuli (positive words; negative words) mixed-design ANOVA was conducted with attentional processing measures as dependent variable. Second, to analyze interpretation bias index as well as in the psychological measures (i.e., reappraisal, rumination, anxiety and depression levels), a series of 2 Group (OCAT; Control) x Time (Time 1: pre-training; Time 2: post-training) mixed-design ANOVAs was conducted. When significant interactions were found, Bonferroni post-hoc comparisons were performed correcting alpha p-values for multiple comparisons.

Finally, exploratory analyses were conducted to test the influence of cognitive bias changes as a response of cognitive training to account for training effects in transfer psychological measures (see Sanchez et al., 2016). First, we computed delta change scores, subtracting T1 (i.e., pre-training) levels from T2 (i.e., post-training) levels for each variable. Second, we conducted a series of bivariate correlations to analyze the relation between delta change scores of attention and interpretation measures, and delta change scores of emotion regulation, and symptom variables. When significant correlations were found, mediation models were conducted following Hayes’ (2013) guidelines (i.e., 5000 bootstrap method with a 95% confidence interval) to further test whether indirect effects of OCAT on far-transfer psychological changes (i.e., emotion regulation, symptoms) would be accounted by close-transfer effects on cognitive bias changes (Sanchez et al., 2016).

## Results

### Group characteristics

Analyses of demographic characteristics showed that there were no differences between groups on age, (*t*(43) = 0.233; *p* = 0.817), or gender (*X*^2^ = 0.012; *p* = 0.913). Additionally, independent samples t-tests were carried out to analyze differences between groups at Time 1. Analyses showed that there were no differences between groups in time attending to both positive (*t*(44) = 0.772; *p* = 0.444) and negative stimuli (*t*(44) = 1.64; *p* = 0.109), interpretation bias (*t*(44) = 1.66; *p* = 0.103), general anxiety (*t*(44) = 0.983; *p* = 0.331), brooding rumination (*t*(44) = 1.68; *p* = 0.103) or reappraisal scores (*t*(44) = 0.161; *p* = 0.873). However, results revealed that, at Time 1, participants in the control group, compared to their counterparts in the active OCAT group, showed higher levels of depression (*t*(44) = 2.54; *p* = 0.015; *d* = 0.75) (see Table 2).

**Table 2:** Mean and standard deviation of dependent variables for each group (OCAT and control) at each assessment time (Time 1; Time 2)

	OCAT (*N* = 22)		Control group (*N* = 24)	
	Time 1 M* (SD)*	Time 2 M* (SD)*	Time 1 M* (SD)*	Time 2 M* (SD)*
Total attention time				
Toward positive stimuli (ms)	3044.5 (1084.04)	2189.3 (1219.6)	3262.3 (821.92)	2604 (1043.5)
Toward negative stimuli (ms)	2774 (919.23)	1764.5 (845.6)	2368.5 (737.43)	1948 (898)
Interpretation bias index (proportion)	.83 (.18)	.92 (.11)	.72 (.24)	.65 (.25)
Depressive symptoms (CESD; range: 0–24)	13.82 (2.24)	14.28 (3.27)	16.17 (3.77)	17.62 (4.1)
Generalized Anxiety symptoms (GAD; range: 0–21)	12.27 (3.10)	12.82 (3.29)	13.17 (3.06)	15.87 (4.4)
Brooding rumination (RRS; range: 5–20)	9.5 (2.34)	8.41 (2.34)	10.75 (2.71)	11.09 (2.8)
Reappraisal (CERQ; range: 4–20)	13.82 (4.32)	13.82 (4.34)	13.62 (3.81)	13.62 (3.8)

In the attention and interpretation bias indices, scores higher than .50 represent a bias towards positive information

*M* mean; *SD* standard deviation

### Effects of the OCAT on cognitive bias change

**Fig. 6:**
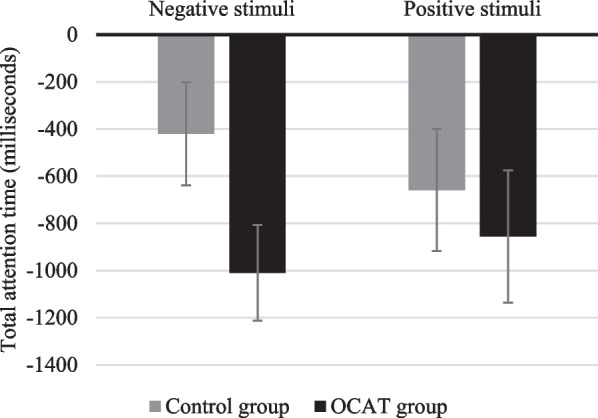
Delta change scores on total time attending towards positive and negative information. Note: Error bars represent the standard error of the mean

*Attentional processing* The 2 Group (OCAT; Control) × 2 Time (Time 1; Time 2) × 2 Type of stimuli (Positive; Negative) revealed a non-significant main effect of group *F*(1, 44) = 0.220; *p* = 0.642, but significant effects of Time *F*(1, 44) = 19.84; *p* < 0.001; *η*_*p*_^*2*^ = 0.311 and Type of stimuli, *F*(1, 44) = 149.21; *p* < 0.001; *η*_*p*_^*2*^ = 0.772, which were qualified by a significant three-way Group x Time x Type of Stimuli interaction *F*(1, 44) = 4.66; *p* = 0.036; *η*_*p*_^*2*^ = 0.096. Post-hoc comparison revealed that both in the active training and the control condition participants spent significantly more time attending to positive than to negative information both at Time 1 and Time 2 (all *p’s* < 0.002). When comparing both groups, post-hoc analyses showed that participants in the control and the training groups did not differ in the time they spent attending to the negative and the positive information, neither at Time 1 (*p* = 0.105, and *p* = 0.444, respectively) nor at Time 2 (*p* = 0.481, *p* = 0.221, respectively). As for pre-post changes, both groups reduced, from Time 1 to Time 2, the time spent attending to both negative (Control Group: *p* = 0.049; OCAT group: *p* < 0.001) and positive information (Control Group: *p* = 0.016; OCAT group: *p* = 0.003). The 3-way interaction was accounted for by group differences in the magnitude of change of their times attending to negative information. Comparisons of delta scores (modeling the interaction effect as difference scores to visualize change and its effect size) for total attention times towards negative and positive stimuli revealed that the decrease of the time attending to stimulus was larger in the OCAT group than in the Control group; for the negative stimuli; *t*(44) = 1.96, *p* = 0.050, *d* = 0.58, but not for the positive stimuli; *t*(44) = 0.517, *p* = 0.608 (see Fig. 6).

**Fig. 7:**
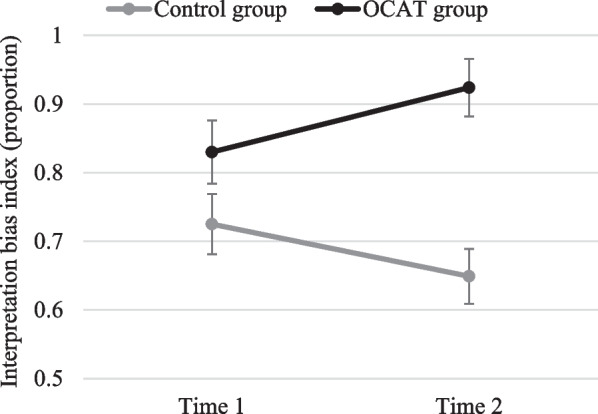
Interpretation bias index before and after the OCAT. Note: Error bars represent the standard error of the mean

*Interpretation bias index* Results revealed a non-significant main effect of Time, *F*(1, 44) = 0.096; *p* = 0.758; but a significant main effect of Group, *F*(1, 44) = 12.53; *p* = 0.001; *η*_*p*_^*2*^ = 0.222, accounted for by a significant Group x Time interaction *F*(1, 44) = 9.24; *p* = 0.004; *η*_*p*_^*2*^ = 0.174. Post-hoc comparisons revealed that, although both groups showed no differences on their interpretation biases at Time 1 (*p* = 0.103), the OCAT group showed higher levels of positive interpretation bias at Time 2 than the Control group (*p* < 0.001). These differences at Time 2 were explained by a significant increase of positive interpretation bias in the OCAT group from Time 1 to Time 2 (*p* = 0.025), whereas the Control group showed a marginally significant decrease (*p* = 0.055) (see Fig. 7).^2^

### Transfer effects to psychological measures

**Fig. 8:**
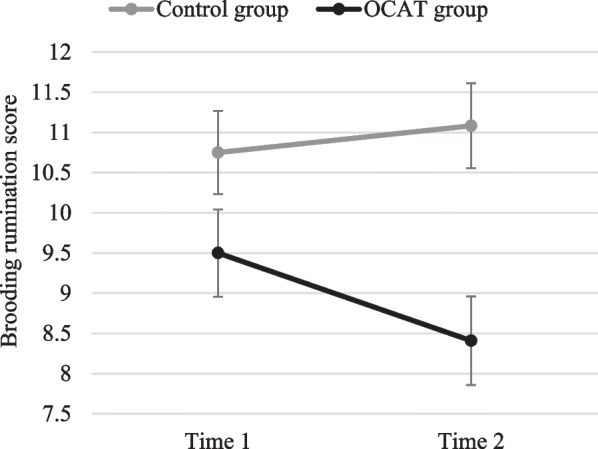
Brooding scores before and after the OCAT. Note: Error bars represent the standard error of the mean

*Brooding rumination* There was no significant main effect of Time, *F*(1, 44) = 1.53; *p* = 0.222; *η*_*p*_^*2*^ = 0.034. A significant main effect of Group, *F*(1, 44) = 8.02; *p* = 0.007; *η*_*p*_^*2*^ = 0.154, was qualified by a significant Group x Time interaction, *F*(1, 44) = 5.41; *p* = 0.025; *η*_*p*_^*2*^ = 0.110. Post-hoc comparisons revealed that, although there were no differences between groups at Time 1 (*p* = 0.103), participants in the OCAT group reported lower levels of brooding rumination at Time 2 than their counterparts in the control group (*p* = 0.001). Also, the OCAT group reported a significant decrease of brooding rumination from Time 1 to Time 2 (*p* = 0.018), which was not observed in the control group (*p* = 0.435; see Fig. 8).

*Reappraisal* Results showed no significant main effects of Group, *F*(1, 44) = 0.010; *p* = 0.919; or Time, *F*(1, 44) = 0.851; *p* = 0.361; or a significant Group × Time interaction effect, *F*(1, 44) = 0.041; *p* = 0.841.

**Fig. 9:**
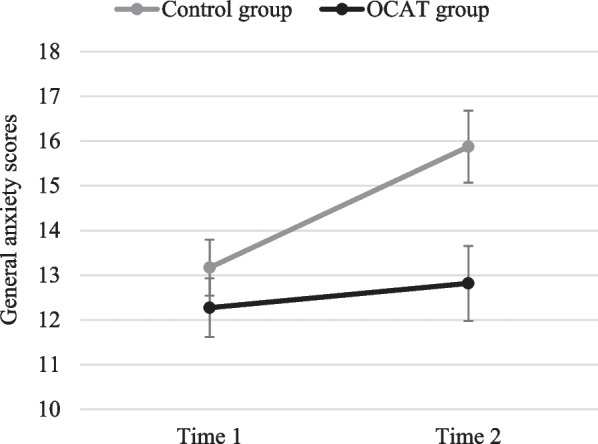
General anxiety scores before and after OCAT. Note: Error bars represent the standard error of the mean

*General anxiety levels* Results showed a significant main effect of Group, *F*(1, 44) = 4.53; *p* = 0.039; *η*_*p*_^*2*^ = 0.093; and Time, *F*(1, 44) = 11.66; *p* = 0.001; *η*_*p*_^*2*^ = 0.210. These main effects were qualified by a significant Group x Time interaction, *F*(1, 44) = 5.15; *p* = 0.028; *η*_*p*_^*2*^ = 0.105. Post-hoc comparisons revealed that, although there were no differences between groups at Time 1 (*p* = 0.331), participants in the OCAT group reported lower levels of anxiety at Time 2 than their counterparts in the control group (*p* = 0.012). Importantly, this between-group difference was accounted by the following within-group patterns: whereas the OCAT group did not report any change on anxiety levels as final exams approached, from Time 1 to Time 2 (*p* = 0.432), the control group showed a significant increase in anxiety across this time period (*p* < 0.001; see Fig. 9).

*Depression levels* Results showed a significant main effect of Group, *F*(1, 44) = 9.97; *p* = 0.003; *η*_*p*_^*2*^ = 0.185. Overall, participants in the control group reported higher levels of depression than their counterparts in the training group. Also, a significant main effect of Time, *F*(1, 44) = 4.24; *p* = 0.045; *η*_*p*_^*2*^ = 0.088, was found. Both groups, increased their levels of depression from Time 1 to Time 2. No significant Group × Time interaction was found, *F*(1, 44) = 1.17; *p* = 0.286.

### Exploratory analyses of relations between in-training cognitive and symptom changes

Analyses showed that larger decreases in the time attending to negative words were significantly related to larger decreases in participants’ depression levels from pre- to post-training (*r* = 0.389; *p* = 0.008). No other significant relations were found (see full details in Additional file 1: Appendix C). Following this pattern of correlations, a bootstrapping mediation model using training group as a predictor, change in total time attending to negative words (delta score) as a mediator, and change in depression levels (delta score) as the dependent variable was carried out. No indirect effect was found (*IC* = − 1.5657; 0.08864).

## Interim discussion

The aim of this first pilot study was to analyze the efficacy of OCAT on targeting attention and interpretation biases as well as its transfer effects to emotion regulation and emotional symptom levels in an unselected sample of undergraduate students who were about to confront a naturalistic stressor (i.e., beginning of final exams). Our results partially supported our hypotheses.

First, we hypothesized that, after the training, participants in the OCAT group would show a larger attentional processing of positive information than their counterparts in the control group. Our results revealed that, after the training, there were no differences between groups in the time they spent attending towards positive and negative information. However, analyses of specific patterns of change showed that participants in the OCAT group specifically decreased their attention to negative information to a larger extent than participants in the control group. Previous research has shown that the relation of attention biases towards negative information and emotional symptomatology follows a gradient (e.g., higher negative attention bias, higher depressive symptomatology—see Blanco et al., 2019). Our results support this idea: decreases in the time attending to negative information was positively related to decreases in depressive symptom levels from pre- to post-training. Overall, results show that OCAT compared to control reduced attention to negative information, which might have a protective effect on depression levels when facing a naturalistic stressor. Second, regarding interpretation biases, it was expected that, after the training, participants in the OCAT group would show a larger positive interpretation bias than their counterparts in the control group. OCAT training resulted in an increased proportion of positive interpretations from pre- to post-training, in comparison to control participants. Taken together, these results highlight that the 10-session OCAT was effective in modifying attentional and interpretation processes.

In terms of transfer to emotion regulation, our hypotheses stated that, after the training, OCAT participants (in comparison to the control participants) would report lower use of rumination, and higher use of reappraisal. Our results revealed that OCAT group indeed showed a significant reduction of brooding rumination after training. This result is in line with previous evidence found using the eye-tracking and computer-based variants of this training (see Sanchez et al., 2016; Sanchez-Lopez et al., 2019a, 2019b, 2019c) where trained participants reported less use of state brooding rumination during an emotion regulation task. The present results are novel, as they support that OCAT on a daily basis may transfer to changes in trait measures of habitual use of rumination. In contrast, no differences were found in the habitual use of reappraisal, which highlights the possibility the OCAT transfer effects to habitual emotion regulation might specifically improve maladaptive forms of regulation when used under conditions of high ecological stress.

Regarding transfer effects to emotional symptoms, in line with our prediction, there were group differences after training in levels of anxiety in the face of naturalistic stress. The control group showed a significant increase of their levels of anxiety from Time 1 to Time 2, whereas these patterns of increased symptom levels in the face of naturalistic stress were absent in the training group. Previous experimental research has proposed that attention bias modification paradigms could act as a vaccine against stressful situations (Browning et al., 2012). Therefore, it is plausible to hypothesize that OCAT training, to the extent to which it is effective to treat cognitive biases and to improve emotion regulation, would act as a buffer leading to differences between groups in their emotional reactions to the approaching stressor (i.e., exam period). Nonetheless, further research would be needed to extend and replicate this pattern of findings.

In sum, the results of this first study show preliminary evidence regarding the efficacy of the OCAT procedure to modify specific components of attention (i.e., total time attending negative information) and interpretation biases. We also observed transfer effects to emotion regulation and symptom levels under stress conditions. In a second study we sought to replicate these findings and to extend them when considering OCAT efficacy for individuals from the general population in the face of a major stressor.

## Study 2. Validation and effectiveness of OCAT to target cognitive biases, emotion regulation and psychological symptoms during COVID-19 lockdown in a community sample

The beginning of COVID-19 pandemic comprised a significant major stressor for the general population, resulting in increased rates of depression and anxiety (Shevlin et al., 2020; Valiente et al., 2021). Recent research, conducted in the context of the COVID-19 lockdown situation of early 2020, has shown that people characterized by more negative interpretation and attention biases reported higher levels of depression and anxiety during the lockdown period (Blanco et al., 2021). Therefore, training attention and interpretation biases emerged as a clear target to improve emotion regulation and emotional symptom levels in the context of the COVID-19 lockdown. The aim of this second proof-of-principle study was to examine the effectiveness of the OCAT on targeting attention and interpretation biases as well as its transfer effect to emotion regulation and symptom levels in a community sample undergoing the COVID-19 lockdown situation. Based on the results of study 1, we hypothesized that participants in the active OCAT condition, in comparison to the control condition, would show training-related changes in attention and interpretation biases as well as transfer effects in their levels of rumination and anxiety.

## Method

### Participants

Fifty-eight participants took part in the study voluntarily. They were recruited by advertising on social media and social networks. As in Study 1, participants were randomly allocated to the active OCAT and the control sham-training group through a simple randomization procedure. Six participants of the active OCAT group, and 4 of the control sham-training group, were excluded from analyses due to dropout and/or technical issues. The final active OCAT group was composed of 23 participants (83% female; mean age = 36.78 years—*SD* = 17.10) and 25 participants were included in the control sham-training group (88% female; mean age = 29.32 years—*SD* = 13.65) (see Additional file 1: Appendix C for full details in the flow diagram of recruitment and participation).

### General procedure

The general procedure was identical to study 1, except for the following minor differences. Due to the COVID-19 lockdown restrictions, participants were contacted through advertising in social media and social networks, and the entire procedure was conducted online. Thus, after participants were randomized to the OCAT or the sham-control group, they were provided with online instructions on how to complete the psychological and emotion regulation measures as well as on how to download, install, and use the mobile training app. First, participants signed an online consent form, before completing the psychological measures via Qualtrics software. Then, they installed the app and completed attention and interpretation biases as baseline assessment (Time 1). Immediately afterwards, they started the first online session of the OCAT or sham-control. Identically to study 1, they then completed a total of 10 sessions of online training, on a daily basis, with the same instructions as in Study 1. As in Study 1, the post-training cognitive bias assessments (Time 2) were collected immediately after completing the last training session, whereas the post-training psychological assessments were collected the day after the last training session via Qualtrics. Given the changing situation experienced across the initial COVID-19 lockdown period, it was decided to restrict recruitment for the study to a specific short-time period during lockdown. Sample recruitment was specifically performed during one week at the end of April 2020 (a very restrictive period of the lockdown in Spain). The study was conducted in compliance with the Declaration of Helsinki (World Medical Association, 2013) and approval from the University Ethical Committee was obtained.

### Experimental task and materials

Experimental task and materials were the same as in study 1.

### Attention and interpretation dependent variables

Attention and interpretation were assessed in the same manner as in study 1. Further, reliability analyses were conducted on the measures in the current study. As in study 1, the results showed excellent internal consistencies of both attention and interpretation bias measures at Time 1 and Time 2 (see Table 3).

**Table 3:** Internal consistency for each scale and cognitive bias measure at each assessment point

	Time 1	Time 2
Attention measures (α)		
Total time attending positive words	.87	.92
Total time attending negative words	.84	.96
Interpretation biases (λ)	.91	.92
Depression (CESD)—(α)	.82	.86
Generalized anxiety (GAD)—(α)	.91	.86
Brooding rumination (RRS)—(α)	.76	.86
Reappraisal (ERQ)—(α)	.81	.83

α = Cronbach’s alpha; λ = Guttman split-half coefficient

### Psychological measures

Given the null finding for reappraisal transfer in study 1, reappraisal was assessed using a different reappraisal measure, the reappraisal subscale of the Emotion Regulation Questionnaire (ERQ—Gross & John, 2003). This served to discard that previous null effects in reappraisal were due to specific characteristics of the measure chosen to assess the construct. The reappraisal ERQ subscale evaluates the use of reappraisal strategy through five Likert-scale items (from 1—*totally agree* to 7—*totally disagree*). In the validation study, it has shown that the reappraisal subscale had a good internal consistency (α = 0.79; Gross & John, 2003).

The rest of the self-reported variables (i.e., depression, anxiety, and brooding rumination) were assessed using the same instruments as in the study 1. In all cases, internal consistencies of the instruments in the study were good (see Table 3).

### Data analysis plan

The data analysis plan followed in the present study was identical to the one used in the study 1.^3^

## Results

### Group characteristics

Analyses of demographic characteristics showed no differences between groups on age, (*t*(45) = 1.66; *p* = 0.104), or gender (*X*^2^ = 0.28; *p* = 0.597). Additionally, independent samples t-tests were carried out to analyze differences between groups at Time 1 on each dependent variable. Results showed that, at Time 1, the training group spent more time attending towards both the positive (*t*(46) = 2.901; *p* = 0.006; *d* = 0.82) and negative stimuli (*t*(46) = 3.231; *p* = 0.003; *d* = 0.94) than their counterparts in the control group. There were no significant differences between groups on any other dependent variables (all *ps* > 0.240; see Table 4).

**Table 4:** Mean and standard deviation on dependent variables for each group (OCAT and control) at each assessment time (Time 1; Time 2)

	OCAT (*N* = 23)		Control group (*N* = 25)	
	Time 1 M (SD)	Time 2 M (SD)	Time 1 M (SD)	Time 2 M (SD)
Total attention time				
Toward positive stimuli (ms)	2527.56 (927.03)	1813.25 (915.93)	1914.96 (549.30)	1527.16 (550.82)
Toward negative stimuli (ms)	2432.04 (853.9)	1422.91 (420.06)	1776.96 (513.99)	1466.67 (557.89)
Interpretation bias index (proportion)	.61 (.29)	.92 (.18)	.54 (.29)	.49 (.31)
Depressive symptoms (CESD; range: 0–24)	12.10 (4.73)	11.00 (5.57)	12.92 (4.64)	12.08 (4.36)
Generalized anxiety symptoms (GAD; range: 0–21)	10.55 (6.03)	9.39 (5.15)	9.60 (5.23)	9.52 (4.10)
Brooding rumination (RRS; range: 5–20)	11.41 (3.97)	11.26 (4.42)	11.40 (3.46)	11.48 (4.13)
Reappraisal (ERQ; range: 7–35)	11.68 (3.68)	13.09 (3.84)	12.00 (3.46)	12.68 (3.54)

In the attention and interpretation bias indices, scores higher than .50 represent a bias towards positive information

*M* mean; *SD* standard deviation

### Effects of the OCAT on cognitive bias measures

#### Attentional processing

**Fig. 10:**
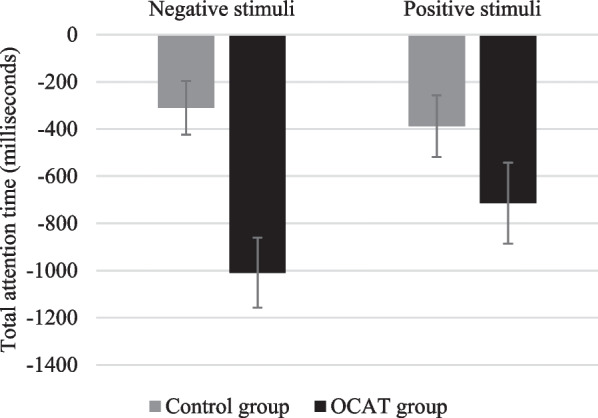
Delta change scores on total time attending towards positive and negative information

As in Study 1, a 2 Group (OCAT; Control) × 2 Time (Time 1; Time 2) × 2 Type of stimuli (Positive; Negative) mixed-design ANOVA showed significant effects of Time *F*(1, 45) = 11.62, *p* < 0.001, *η*_*p*_^*2*^ = 0.205 and Type of stimuli, *F*(1, 45) = 46.59, *p* < 0.001, *η*_*p*_^*2*^ = 0.509, qualified by a significant two-way Group x Type of Stimuli, *F*(1, 45) = 8.35, *p* = 0.006, *η*_*p*_^*2*^ = 0.157, and a trend significant three-way Group x Time x Type of Stimuli interaction *F*(1, 45) = 3.89, *p* = 0.055, *η*_*p*_^*2*^ = 0.080. Post-hoc comparison showed that, at Time 1, participants in the control group attended significantly more to positive stimuli than to negative stimuli (*p* = 0.007). However, at Time 2, there were no differences in the control group in their time attending to positive or negative stimuli (*p* = 0.637). In the case of the training group, there were no differences in the time they spent attending to positive stimuli comparing to negative stimuli at Time 1 (*p* = 0.062) whereas at Time 2, they attended significantly more to positive stimuli than to the negative stimuli (*p* = 0.004). Post-hoc comparisons also showed that the control group reduced their time attending to both positive (*p* = 0.013) and negative stimuli (*p* = 0.021) from Time 1 to Time 2. The same pattern was also found for the training group. Trained participants also reduced the time they attended to the positive (*p* < 0.001) and negative stimuli (*p* < 0.001) from Time 1 to Time 2. As in Study 1, analyses of differences between groups in the magnitude of changes revealed that the 3-way interaction was accounted for by group differences in the magnitude of change of their times attending to negative information: whereas there were no differences between groups in the magnitude of change for the time attending to positive information; *t*(45) = 1.52; *p* = 0.136; the reduction of the time attending to negative stimuli was significantly larger for the training than for the control group; *t*(45) = 3.76; *p* < 0.001; *d* = 0.55 (see Fig. 10).

**Fig. 11:**
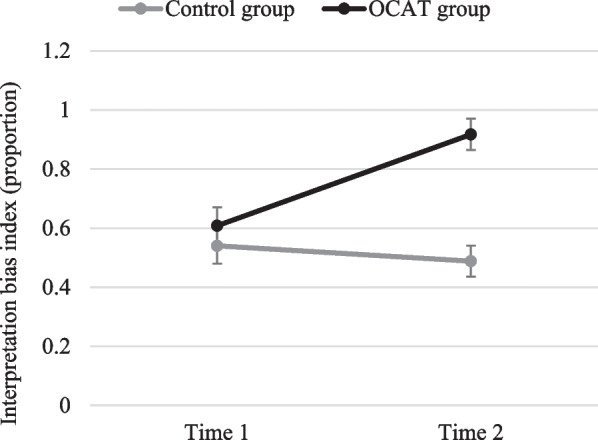
Interpretation bias index before and after the OCAT for each group

*Interpretation bias index* Analyses showed significant main effects of Group, *F*(1, 45) = 11.95; *p* = 0.001;* η*_*p*_^*2*^ = 0.210, and Time, *F*(1, 45) = 12.48; *p* = 0.001; *η*_*p*_^*2*^ = 0.217, qualified by a significant Group x Time interaction, *F*(1, 45) = 24.63; *p* < 0.001; *η*_*p*_^*2*^ = 0.354. Post-hoc comparisons showed that, whereas there were no statistical differences between groups at Time 1 (*p* = 0.435), the training group showed a higher positive interpretation bias at Time 2 compared to the control group (*p* < 0.001). The control group did not show any change in positive attention bias from Time 1 to Time 2 (*p* = 0.312), whereas the training group showed a significant increase in positive interpretation bias from Time 1 to Time 2 (*p* < 0.001; see Fig. 11).

#### Transfer effects to psychological measures

*Brooding rumination* No significant effects of Group, *F*(1, 45) = 0.001; *p* = 0.972, Time, *F*(1, 45) = 0.046; *p* = 0.832, or Group × Time, *F*(1, 45) = 0.003; *p* = 0.956, were found.

*Reappraisal* Analysis revealed no significant main effect of Group, *F*(1, 45) = 0.005; *p* = 0.942. Also the Group x Time interaction was not significant, *F*(1, 45) = 0.623; *p* = 0.434. However, a main effect of Time, *F*(1, 45) = 4.73; *p* = 0.035; *η*_*p*_^*2*^ = 0.095, emerged from the analysis. Post-hoc comparisons showed that participants in general reported a significant increase of the use of reappraisal from Time 1 to Time 2 (*p* = 0.035).

*General anxiety* Analysis revealed no significant main effects of Group, *F*(1, 45) = 0.161; *p* = 0.690, Time, *F*(1, 45) = 0.660; *p* = 0.421, or a significant Group x Time interaction, *F*(1, 45) = 0.455; *p* = 0.503.

*Depression* No significant main effects of Group, *F*(1, 45) = 0.451; *p* = 0.505, Time, *F*(1, 45) = 2.19; *p* = 0.146, or a significant Group x Time interaction, *F*(1, 45) = 0.003; *p* = 0.954, emerged on the analysis.

#### Exploratory analyses of relations between in-training cognitive and symptom changes

Pearson correlation analysis is showed that trained-related changes in positive interpretation bias were negatively related to changes in anxiety levels (*r* = -0.327; *p* = 0.027) and in the use of brooding rumination (*r* = -0.292; *p* = 0.049). No other significant relations were found (see Additional file 1: Appendix D material for full details). Based on the previously reported correlations, we conducted two separate mediation models using training group as the predictor variable, interpretation bias change (delta score) as the mediator, and rumination and anxiety change scores (delta scores) as dependent variables. Regarding the first model (i.e., group → interpretation bias change → anxiety level change), the analysis showed that whereas neither the total effect (*CI* = − 3.2475; 1.5202) nor the direct effect (*CI* = − 1.8111; 3.8447) were statistically significant, a significant indirect effect was supported (*CI* = − 4.2138; − 0.0245), showing a partially standardized indirect effect of − 0.47. Regarding the second model (i.e., group → interpretation bias change → brooding rumination change) the same pattern of results was revealed. No significant total (*CI* = − 1.9884; 2.1778) or direct (*CI* = − 0.4637; 4.3910) effect was found but a significant indirect effect was supported (*CI* = − 3.8171; − 0.2019), with a partially standardized indirect effect of − 0.54 (see Table 5). Thus, larger improvements in positive interpretation bias as result of receiving OCAT resulted in larger reduction of brooding rumination and anxiety symptom levels, with decreases of 0.54 and 0.47 standard deviations respectively.

**Table 5:** Bootstrap mediation analysis

Path/effect	*B* mean indirect effect	SE of mean	95% CI mean indirect effect	
			Lower	Upper
Group → interpretation bias (DS) → anxiety (DS)				
*a.* Group → interpretation bias (DS)	0.3618	0.0744	0.2119	0.5117*
*b.* Interpretation bias (DS) → anxiety (DS)	− 5.1973	2.2919	− 9.8194	− 0.5753*
*c.* Group → anxiety (DS)	− 0.8636	1.1828	− 3.2475	1.5202
*c'*	1.0168	1.4022	− 1.8111	3.8447
*a* × *b*	− 1.8804	1.0931	− 4.2138	− 0.0245*
Group → interpretation bias (DS) → rumination (DS)				
*a.* Group → interpretation bias (DS)	0.3618	0.0744	0.2119	0.5117*
*b.* Interpretation bias (DS) → rumination (DS)	− 5.1657	1.9672	− 9.1330	− 1.1984*
*c.* Group → rumination (DS)	0.0947	1.0336	− 1.9884	2.1778
*c'*	1.9637	1.2036	− 0.4637	4.3910
*a* × *b*	− 1.8690	0.9266	− 3.8171	− 0.2019*

*DS* delta score; c = total effect; *c'* = direct effect; *a* × *b* = indirect effect

*Zero is not in the 95% confidence interval (indirect effect significantly different from zero at *p* < .05)

### Interim discussion

Previous research has shown that attention and interpretation biases play a major role in psychological adjustment during COVID-19 lockdown (Blanco et al., 2021). Therefore, the aim of this second study was to extend and replicate findings from Study 1, assessing the efficacy of the OCAT to modify both attention and interpretation biases, as well as its transfer effects to emotion regulation and emotional symptoms, during the COVID-19 lockdown experienced during 2020. It was hypothesized that participants in the OCAT group would show, in comparison with control participants, changes in attention and interpretation processing. Our results partially supported this hypothesis. First, regarding attentional processing, our results revealed that OCAT participants showed a significantly higher reduction in the total time attending to negative information in the active compared to control group from pre- to post-training, as in study 1. Second, regarding interpretation bias, also replicating findings from study 1, trained participants showed significant increases, from Time 1 to Time 2, on positive interpretations, whereas the control group did not. Thus, and overall, the training was effective in modifying information processing biases within a highly stressful context.

Following the results of Study 1, we also hypothesized that training-related improvements in attention and interpretation biases would transfer to improved rumination and anxiety levels. Different to Study 1, groups did not differ in emotion regulation or symptom levels from pre- to post-training. This could be accounted by a larger heterogeneity in the characteristics of the community sample tested in Study 2. Furthermore, the sample of the present study was undergoing a major stressor when starting the study, whereas the sample of Study 1 was anticipating an upcoming stressor. The different results found in each study suggest the possibility that this type of CBM trainings act as stress buffer (Browning et al., 2012) reducing its efficacy when the stressor is already present. Future research should address this relevant issue by comparing training effects for comparable similar types of stressors when they are anticipated vs. when they are already being confronted. However, although exploratory, further analyses using mediation models showed that OCAT training did reduce both brooding rumination and anxiety levels, as hypothesized, but indirectly via training-based generated changes in positive interpretation bias. Thus, larger individual increases on positive interpretations following active OCAT accounted for larger decreases in both rumination and anxiety levels.

In sum, the results of this second study further supported the efficacy of the OCAT procedure to modify both attention and interpretation processing during the occurrence of a major stressor and point out a potential transfer to emotion regulation and symptom levels via cognitive bias modification.

## General discussion

Cognitive processing (e.g., attention and interpretation) of emotional information have been postulated as crucial variables implicated in the onset and maintenance of diverse forms of psychopathology such as depression and anxiety (Mathews & MacLeod, 2005). Despite research efforts to develop procedures to modify maladaptive functioning in these processes, the efficacy of initial ABM procedures has been questioned (Cristea et al., 2015; Fodor et al., 2020). Further promising training approaches, such as ECAT or MCAT (see Sanchez et al., 2016, Sanchez-Lopez et al., 2019a, 2019b) still present limitations in terms of their feasibility to be implemented out of laboratory settings. Therefore, the general aim of the present research was to develop and test the efficacy of a new online variant of this training procedure, the online contingent attention training (developed as a mobile application).

Overall, the results of both studies show that the OCAT app is effective to modify attention and interpretation processes and that these changes seem to transfer to reductions of habitual use of brooding rumination and buffer against anxiety in the face of naturalistic stressors. The OCAT smartphone app was developed based on the same training principles of original ECAT and MCAT procedures (Sanchez et al., 2016; Sanchez-Lopez et al., 2019a) and our results across the two studies were mostly in line with those previously found in previous ECAT and MCAT studies. Additionally, as shown in the analyses of internal consistency of attention and interpretation biases measures, the reliability of these assessments was excellent. Taken together, this evidence supports the idea that OCAT app might be a promising alternative to remotely target maladaptive attention and interpretation processes, highlighting the ability of the mobile app to promote positive information processing biases, adaptive emotion regulation and resilient functioning in response to stress.

However, despite the promising results of these proof-of-principle studies, inconsistencies were found between Study 1 and Study 2, and future research should address them. For instance, whereas in Study 1 group-based transfer effects to brooding rumination and anxiety levels were found; in Study 2 these transfer effects emerged only from individual-based exploratory indirect effect models. As explained above, these differences could be accounted by the different nature of the stressors (the exam period in Study 1 vs. the COVID-19 lock-down in Study 2) as well as by differences in the sample characteristics (undergraduate students in Study 1 vs. community sample in Study 2). Additionally, there is still scarce evidence regarding the optimal number of sessions for these types of trainings. Future studies should address the effective-optimal dose of OCAT to maximize emotional improvements resulting from its use during periods conveying major stress conditions (see Vazquez et al., 2016). In the present studies, a dose of 10 sessions over two weeks was used. However, other studies have applied ABM trainings for different time periods ranging from one single session to several weeks, showing mixed results (Haeffel et al., 2012).

Other issues remain also unclear and should be analyzed in further pre-registered randomized control trials. For instance, the training provides attention-contingent feedback to train participants across each trial. This feedback is thought to enhance participants’ awareness of their attentional patterns, which would also facilitate the use of top-down cognitive strategies to regulate attention (Bernstein & Zvielli, 2014). It is well-know that impairments in cognitive control and executive functions are often observed in emotional disorders (Meiran et al., 2011) and can influence symptoms through increasing the use of maladaptive emotion regulation strategies such as rumination (Joormann & Vanderlind, 2014). It is thus possible that the effectiveness of the OCAT to modify emotional processes would operate through changes in these (internal) cognitive control mechanisms rather than only on the modification of tendencies to attend and interpret emotional information. This opens new venues of research, where the clarification of cognitive control functions targeted by OCAT and of the role of these functions as mediators of training effects in emotional functioning might help to maximize the effects of OCAT through new empirically informed evidence (Goodwin et al., 2019). Also, as commonly done in CBM research field, both attention and interpretation changes were assessing using a similar procedure as is the one used to train participants’ attention and interpretation. It must be noted that this type of approach might produce confounding effects as a result of demand effects of the task. Future research is needed to analyze transfer effects of multisession OCAT to attention and interpretation biases using other different cognitive bias measures in order to control for these potential demand effects as well as to analyze the generalizability of the attention and interpretation changes to other types of measures, as previously done in studies testing transfer effects of this training in a single session format (see for instance, Sanchez-Lopez et al., 2019a, 2019b, 2019c). Furthermore, despite that we conducted additional multilevel models in our studies to test training effects in attention and interpretation biases, which included within-person random effects, we could not further model random terms of time and type of stimuli, due to problems of convergence. Further studies should thus consider using larger samples to replicate that the reported transfer effects due to the training remain evident while also controlling for other sources of variability, such as random effects due to time and to the type of stimuli used.

Despite of these limitations, the OCAT app opens a new venue to implement an online training tool able of modifying attention and interpretation processes and transferring to promote adaptive emotion regulation and stress resilience. Of note, this app is suited to allow reaching large-scale clinically or at-risk populations. This is in line with claims regarding the necessity of theory-driven and empirically informed interventions able to intervene causal mechanism on psychopathology (NIMH, 2011). Further, randomized controlled trials are the next step to demonstrate the efficacy of OCAT. Ultimately, the OCAT may have potential to increase the accessibility to psychological trainings to a large part of the population who need them most (Kazdin & Blase, 2011).

## Supplementary Information

**Additional file 1. Appendix A**. Flow diagram of participant’s recruitment and allocation (Study 1). **Appendix B**. General task procedure. **Appendix C**. Multilevel analyses (Study 1). **Appendix D**. Bivariate correlations between change delta scores of attention and interpretation and change delta scores of emotion regulation and symptoms variables (Study 1). **Appendix E**. Flow diagram of participant’s recruitment and allocation (Study 2). **Appendix F**. Multilevel analyses using within-subject differences as random effects (Study 2). **Appendix G**. Bivariate correlations between change delta scores of the main variables (Study 2).

## Data Availability

The datasets used and/or analyzed during the current study are available from the corresponding author on reasonable request.

## Abbreviations

ABM: Attention bias modification
AB: Attention bias
CESD-8: Center for Epidemiological Studies on Depression-8
ECAT: (Eye)gaze-contingent feedback training
GAD-7: Generalized Anxiety Disorder Scale
MCAT: Mouse-based contingent attentional training
OCAT: Online contingent attention training
SST: Scrambled sentence task

## Footnotes

1 Homoscedasticity was met for all the variables. The assumption of normality was not met for reappraisal and anxiety levels on the training group. However, due to the robustness of ANOVA for normality violations, no further transformations were conducted.

2 In order to control for within-subject differences, multilevel analyses were carried out for attention and interpretation biases using within-subjects differences as random effects in both studies. To construct these models, trials based on the stimuli type (negative, positive) were nested within assessment times (Time1, Time2), then nested within individuals. Analyses were performed in R using the lme function for continuous variables (i.e., attention indices) and the glme function for dichotomous variables (i.e., interpretation index). All models were conducted introducing random terms per individual. Fixed effects for Group x Time x Stimuli were considered. Random slopes and intercepts could not be modelled for Time and Stimuli type due to problems of convergence. All results using this further approach were similar to the ones reported in the main manuscript, using mixed ANOVAs (see Appendix C and F for the full set of results using multilevel models for Study 1 and Study 2, respectively).

3 All variables met assumptions of normality and homoscedasticity in Study 2.
