# Supplementary material for: Online Contingent Attention Training (OCAT): transfer effects to cognitive biases, rumination, and anxiety symptoms from two proof-of-principle studies
Source: Cogn Res Princ Implic. 2023 May 8;8:28. doi: 10.1186/s41235-023-00480-3 (PMC10166036; doi:10.1186/s41235-023-00480-3)
Supplement: Supplementary file 1 — Additional file 1. Appendix A. Flow diagram of participant’s recruitment and allocation (Study 1). Appendix B. General task procedure. Appendix C. Multilevel analyses (Study 1). Appendix D. Bivariate correlations between change delta scores of attention and interpretation and change delta scores of emotion regulation and symptoms variables (Study 1). Appendix E. Flow diagram of participant’s recruitment and allocation (Study 2). Appendix F. Multilevel analyses using within-subject differences as random effects (Study 2). Appendix G. Bivariate correlations between change delta scores of the main variables (Study 2). [file 41235_2023_480_MOESM1_ESM.docx]

*Appendix A.* Flow diagram of participant’s recruitment and allocation (Study 1)

Participants selected (*n*= 64)

Enrollment

Allocation

Allocated to Training (*n*= 32)

Analysis

Lost to (*n* = 10)

- Dropouts (*n*= 6)
- Technical issues (*n*= 2)
- Failure to receive outcome measure package (*n*= 1)
- Outlier (*n* = 1)

Lost to (*n* = 6)

- Dropouts (*n*= 2)
- Technical issues (*n*= 0)
- Failure to receive outcome measure package (*n*= 3)
- Outlier (*n* = 1)

Analyzed (*n*= 24)

Analyzed (*n*= 22)

Allocated to Control (*n*= 32)

Lost (n = 16)

*Appendix B.* General task procedure.


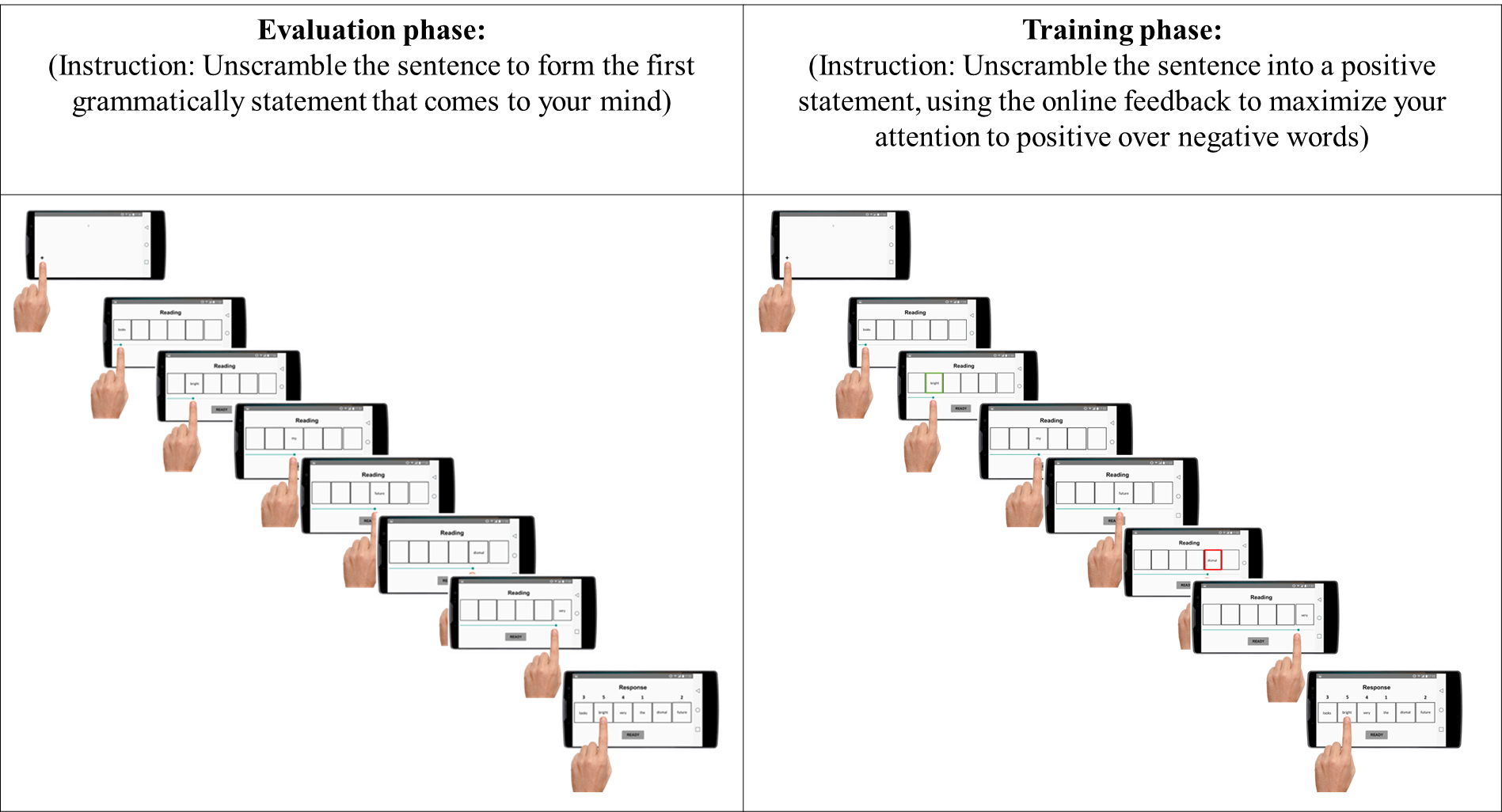


*Appendix C.* Multilevel analyses (Study 1).

1. Model:

- Predictors: Group (0- CTL, 1-OCAT) x Time (1-Time1, 2-Time2) x Stimuli (0-negative, 1-positive)
- Outcome: Attention to emotional word

Linear mixed-effects model fit by maximum likelihood

Data: Data3

AIC BIC logLik

34941.88 34998.2 -17460.94

Random effects:

Formula: ~1 | id

(Intercept) Residual

StdDev: 729.8149 1109.284

Fixed effects: AT ~ 1 + Group * Emo * Assessment_Time

Value Std.Error DF t-value p-value

(Intercept) 2771.0203 211.3680 2011 13.109935 0.0000

Group 1079.5429 309.2535 44 3.490802 0.0011

Emo 1136.8757 211.1045 2011 5.385368 0.0000

Assessment_Time -415.4103 94.9776 2011 -4.373772 0.0000

Group:Emo -1115.7680 310.0363 2011 -3.598830 0.0003

Group:Assessment_Time -613.8958 140.5883 2011 -4.366619 0.0000

Emo:Assessment_Time -259.9179 133.6221 2011 -1.945171 0.0519

Group:Emo:Assessment_Time 492.6561 196.2900 2011 2.509839 0.0122

Correlation:

(Intr) Group Emo Asss_T Grp:Em Gr:A_T Em:A_T

Group -0.683

Emo -0.500 0.342

Assessment_Time -0.671 0.459 0.668

Group:Emo 0.341 -0.502 -0.681 -0.455

Group:Assessment_Time 0.454 -0.679 -0.451 -0.676 0.663

Emo:Assessment_Time 0.474 -0.324 -0.949 -0.704 0.646 0.476

Group:Emo:Assessment_Time -0.323 0.476 0.646 0.479 -0.949 -0.698 -0.681

Standardized Within-Group Residuals:

Min Q1 Med Q3 Max

-3.2648983 -0.6211352 -0.1776288 0.4249335 6.2668535

Based on the significant 3-way interaction, we then conducted separate models for each type of Stimuli (i.e., negative and positive)

- 1. Model:
- Predictors: Group (0- CTL, 1-OCAT) x Time (1-Time1, 2-Time2)
- Outcome: Attention to negative word

Linear mixed-effects model fit by maximum likelihood

Data: Data3

AIC BIC logLik

17382.67 17412.3 -8685.335

Random effects:

Formula: ~1 | id

(Intercept) Residual

StdDev: 643.302 1048.894

Fixed effects: ATneg ~ 1 + Group * Assessment_Time

Value Std.Error DF t-value p-value

(Intercept) 2768.4113 193.37524 983 14.316266 0.000

Group 1000.6159 284.12125 44 3.521792 0.001

Assessment_Time -413.4880 90.13932 983 -4.587210 0.000

Group:Assessment_Time -569.5292 134.22282 983 -4.243162 0.000

Correlation:

(Intr) Group Asss_T

Group -0.681

Assessment_Time -0.695 0.473

Group:Assessment_Time 0.467 -0.704 -0.672

Standardized Within-Group Residuals:

Min Q1 Med Q3 Max

-2.2501355 -0.6311828 -0.1920461 0.4017022 6.2003044

- 1. Model:
- Predictors: Group (0- CTL, 1-OCAT) x Time (1-Time1, 2-Time2)
- Outcome: Attention to positive word

Linear mixed-effects model fit by maximum likelihood

Data: Data3

AIC BIC logLik

17634.63 17664.26 -8811.313

Random effects:

Formula: ~1 | id

(Intercept) Residual

StdDev: 796.0269 1170.62

Fixed effects: ATpos ~ 1 + Group * Assessment_Time

Value Std.Error DF t-value p-value

(Intercept) 3911.085 226.8307 984 17.242308 0.0000

Group -5.917 332.9896 44 -0.017769 0.9859

Assessment_Time -679.063 100.5355 984 -6.754461 0.0000

Group:Assessment_Time -137.794 149.8567 984 -0.919503 0.3581

Correlation:

(Intr) Group Asss_T

Group -0.681

Assessment_Time -0.660 0.450

Group:Assessment_Time 0.443 -0.671 -0.671

Standardized Within-Group Residuals:

Min Q1 Med Q3 Max

-3.0947846 -0.6093676 -0.1941886 0.4282804 5.7588851

1. Model:

- Predictors: Group (0- CTL, 1-OCAT) x Time (1-Time1, 2-Time2)
- Outcome: Interpretation bias index

Generalized linear mixed model fit by maximum likelihood (Laplace Approximation) [glmerMod]

Family: binomial ( logit )

Formula: IB ~ 1 + Group * Assessment_Time + (1 | id)

Data: Data3

AIC BIC logLik deviance df.resid

865.1 889.4 -427.6 855.1 936

Scaled residuals:

Min 1Q Median 3Q Max

-4.2669 0.1420 0.2830 0.4716 1.4244

Random effects:

Groups Name Variance Std.Dev.

id (Intercept) 1.296 1.138

Number of obs: 941, groups: id, 46

Fixed effects:

Estimate Std. Error z value Pr(>|z|)

(Intercept) 1.7000 0.4250 4.000 6.33e-05 ***

Group -0.6731 0.6786 -0.992 0.321263

Assessment_Time -0.4433 0.2173 -2.040 0.041399 *

Group:Assessment_Time 1.3889 0.3988 3.482 0.000497 ***

---

Signif. codes: 0 ‘***’ 0.001 ‘**’ 0.01 ‘*’ 0.05 ‘.’ 0.1 ‘ ’ 1

Correlation of Fixed Effects:

(Intr) Group Asss_T

Group -0.616

Assssmnt_Tm -0.789 0.488

Grp:Assss_T 0.433 -0.809 -0.547

*Appendix D.* Bivariate correlations between change delta scores of attention and interpretation and change delta scores of emotion regulation and symptoms variables (Study 1).

| DV: Delta scores | Interpretation bias | Total attention time (positive) | Total attention time (negative) |
| --- | --- | --- | --- |
| Interpretation bias | 1 |  |  |
| Total attention time (positive) | .202 | 1 |  |
| Total attention time (negative) | .152 | .867** | 1 |
| Depression | .025 | .262 | .389** |
| Anxiety | -.115 | -.084 | .043 |
| Brooding Rumination | -.165 | .063 | .033 |
| Reappraisal | .081 | .023 | .034 |

Note. ** *p* < .01

*Appendix E.* Flow diagram of participant’s recruitment and allocation (Study 2)

Analysis

Lost to (*n* = 6)

- Dropouts (*n*= 3)
- Technical issues (*n*= 3)

Lost to (*n* = 4)

- Dropouts (*n*= 3)
- Technical issues (*n*= 1)

Analyzed (*n*= 25)

Analyzed (*n*= 23)

Lost (n = 10)

Participants selected (*n*= 58)

Enrollment

Allocation

Allocated to Training (*n*= 29)

Allocated to Control (*n*= 29)

*Appendix F.* Multilevel analyses using within-subject differences as random effects (Study 2)

1. Model:

- Predictors: Group (0- CTL, 1-OCAT) x Time (1-Time1, 2-Time2) x Stimuli (0-negative, 1-positive)
- Outcome: Attention to emotional word

Linear mixed-effects model fit by maximum likelihood

Data: Data3

AIC BIC logLik

38024.19 38081.39 -19002.1

Random effects:

Formula: ~1 | id

(Intercept) Residual

StdDev: 705.7821 1082.001

Fixed effects: AT ~ 1 + Group * Emo * Assessment_Time

Value Std.Error DF t-value p-value

(Intercept) 2086.0952 199.53778 2198 10.454638 0.0000

Group 1571.9885 289.08995 46 5.437714 0.0000

Emo 191.7003 198.72012 2198 0.964675 0.3348

Assessment_Time -315.1752 89.83406 2198 -3.508415 0.0005

Group:Emo -403.3821 288.55551 2198 -1.397936 0.1623

Group:Assessment_Time -760.2793 129.81339 2198 -5.856710 0.0000

Emo:Assessment_Time -65.6036 126.45826 2198 -0.518777 0.6040

Group:Emo:Assessment_Time 377.2630 182.92775 2198 2.062361 0.0393

Correlation:

(Intr) Group Emo Asss_T Grp:Em Gr:A_T Em:A_T

Group -0.690

Emo -0.498 0.344

Assessment_Time -0.669 0.462 0.667

Group:Emo 0.343 -0.499 -0.689 -0.460

Group:Assessment_Time 0.463 -0.671 -0.462 -0.692 0.668

Emo:Assessment_Time 0.472 -0.326 -0.948 -0.704 0.653 0.487

Group:Emo:Assessment_Time -0.326 0.473 0.655 0.487 -0.948 -0.705 -0.691

Standardized Within-Group Residuals:

Min Q1 Med Q3 Max

-3.1084907 -0.4594709 -0.1108934 0.2957160 18.5265940

Based on the significant 3-way interaction, we then conducted separate models for each type of Stimuli (i.e., negative and positive)

- 1. Model:
- Predictors: Group (0- CTL, 1-OCAT) x Time (1-Time1, 2-Time2)
- Outcome: Attention to negative word

Linear mixed-effects model fit by maximum likelihood

Data: Data3

AIC BIC logLik

18933.83 18963.99 -9460.916

Random effects:

Formula: ~1 | id

(Intercept) Residual

StdDev: 580.8173 1030.605

Fixed effects: ATneg ~ 1 + Group * Assessment_Time

Value Std.Error DF t-value p-value

(Intercept) 2079.9587 177.68771 1076 11.705698 0e+00

Group 1590.5294 257.62468 46 6.173824 0e+00

Assessment_Time -309.0387 85.85766 1076 -3.599430 3e-04

Group:Assessment_Time -788.3246 123.98283 1076 -6.358337 0e+00

Correlation:

(Intr) Group Asss_T

Group -0.690

Assessment_Time -0.716 0.494

Group:Assessment_Time 0.496 -0.719 -0.692

Standardized Within-Group Residuals:

Min Q1 Med Q3 Max

-2.7877216 -0.4643958 -0.1521791 0.3132286 19.7686820

- 1. Model:
- Predictors: Group (0- CTL, 1-OCAT) x Time (1-Time1, 2-Time2)
- Outcome: Attention to positive word

Linear mixed-effects model fit by maximum likelihood

Data: Data3

AIC BIC logLik

19136.42 19166.58 -9562.211

Random effects:

Formula: ~1 | id

(Intercept) Residual

StdDev: 835.4426 1115.287

Fixed effects: ATpos ~ 1 + Group * Assessment_Time

Value Std.Error DF t-value p-value

(Intercept) 2282.4948 221.6922 1076 10.295784 0e+00

Group 1136.7967 321.1457 46 3.539816 9e-04

Assessment_Time -385.4782 92.9856 1076 -4.145567 0e+00

Group:Assessment_Time -351.3227 134.2454 1076 -2.617018 9e-03

Correlation:

(Intr) Group Asss_T

Group -0.690

Assessment_Time -0.621 0.429

Group:Assessment_Time 0.430 -0.624 -0.693

Standardized Within-Group Residuals:

Min Q1 Med Q3 Max

-3.37434624 -0.45777740 -0.08472316 0.27207925 16.01903603

1. Model:
   - Predictors: Group (0- CTL, 1-OCAT) x Time (1-Time1, 2-Time2)

- Outcome: Interpretation bias index

Generalized linear mixed model fit by maximum likelihood (Laplace Approximation) [glmerMod]

Family: binomial ( logit )

Formula: IB ~ 1 + Group * Assessment_Time + (1 | id)

Data: Data3

AIC BIC logLik deviance df.resid

987.2 1011.6 -488.6 977.2 965

Scaled residuals:

Min 1Q Median 3Q Max

-5.7327 -0.5754 0.2322 0.6017 2.6827

Random effects:

Groups Name Variance Std.Dev.

id (Intercept) 1.908 1.381

Number of obs: 970, groups: id, 48

Fixed effects:

Estimate Std. Error z value Pr(>|z|)

(Intercept) 0.5228 0.4281 1.221 0.221967

Group -2.3739 0.6735 -3.525 0.000424 ***

Assessment_Time -0.2656 0.2087 -1.273 0.202971

Group:Assessment_Time 2.7189 0.3881 7.005 2.47e-12 ***

---

Signif. codes: 0 ‘***’ 0.001 ‘**’ 0.01 ‘*’ 0.05 ‘.’ 0.1 ‘ ’ 1

Correlation of Fixed Effects:

(Intr) Group Asss_T

Group -0.637

Assssmnt_Tm -0.719 0.458

Grp:Assss_T 0.391 -0.754 -0.540

*Appendix G.* Bivariate correlations between change delta scores of the main variables (Study 2).

| DV: Delta scores | Interpretation bias | Total attention time (positive) | Total attention time (negative) |
| --- | --- | --- | --- |
| Interpretation bias | 1 |  |  |
| Total attention time (positive) | -.189 | 1 |  |
| Total attention time (negative) | -.236 | .588** | 1 |
| Depression | -.133 | .074 | .128 |
| Anxiety | -.327* | .078 | .118 |
| Rumination | -.292* | .107 | .098 |
| Reappraisal | .193 | -.089 | -.145 |

Notes. * *p* < .05; ** *p* < .01
